# Supplementary material for: Effects of Pro-Inflammatory Cytokines on Hepatic Metabolism in Primary Human Hepatocytes
Source: Int J Mol Sci. 2022 Nov 28;23(23):14880. doi: 10.3390/ijms232314880 (PMC9740548; doi:10.3390/ijms232314880)
Supplement: Supplementary file 1 [file ijms-23-14880-s001.zip › Suppl. Fig S1.pdf]

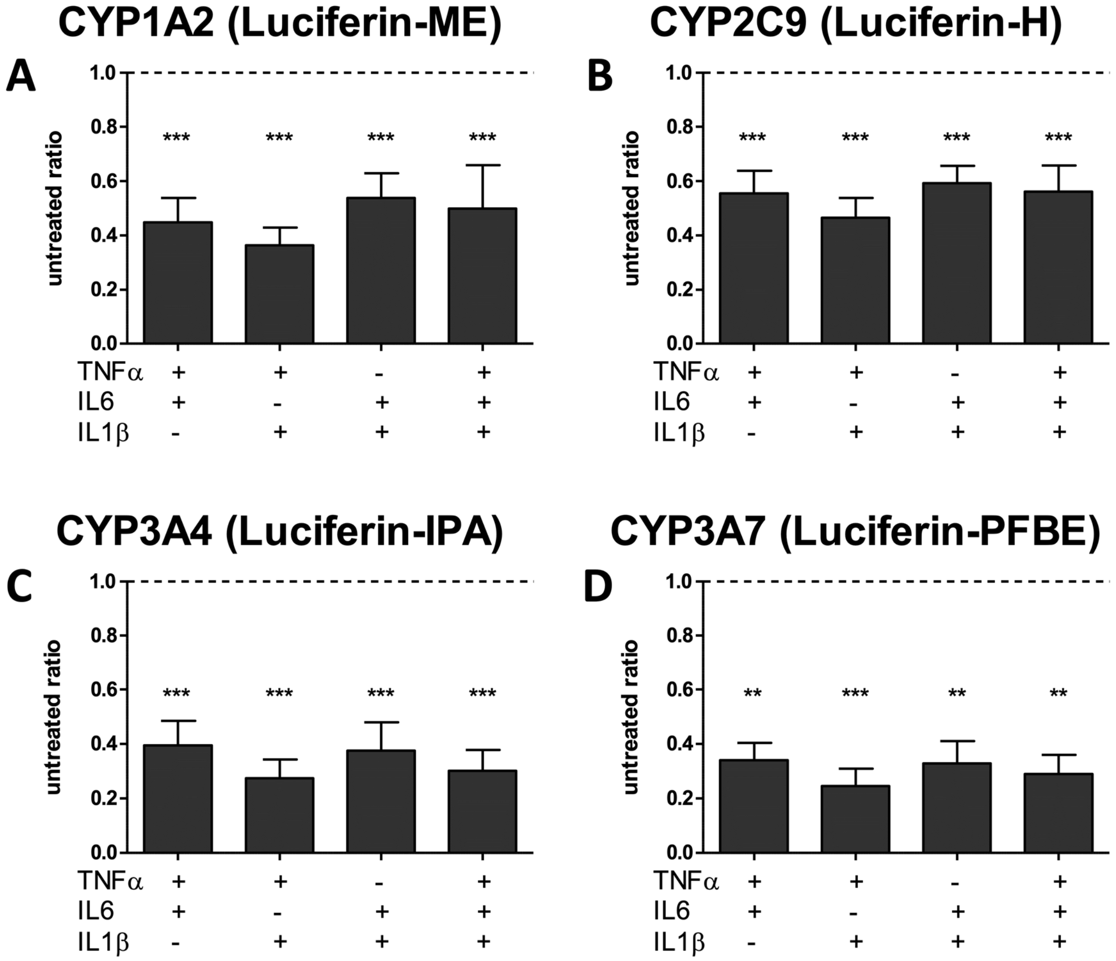

Figure S1. Specific CYP activity inhibitions in human hepatocytes by 72 hours cytokine mixture exposure (10 ng/ml/each) for: (A) CYP1A2; (B) CYP2C9; (C) CYP3A4; (D) CYP3A7. ** $p < 0.001$; *** $p < 0.0001$.
