# Supplementary material for: Effects of Pro-Inflammatory Cytokines on Hepatic Metabolism in Primary Human Hepatocytes
Source: Int J Mol Sci. 2022 Nov 28;23(23):14880. doi: 10.3390/ijms232314880 (PMC9740548; doi:10.3390/ijms232314880)
Supplement: Supplementary file 1 [file ijms-23-14880-s001.zip › Suppl. Table S1.pdf]

Supplementary Table S1. Demographic characteristics for all the donors and resulting cells.

	Category	gender	Age (years)	Viability (%)	Phase 1	Phase 2	Phase 3	Ammonia
1	Primary HyperOxaluria	M	1	91.0	x			x
2	Carbamoyl Phosphate Synthetase-1 deficiency	F	1	90.0	x			x
3	Maple Syrup Urine Disease	F	1.5	78.0				x
4	Focal Nodular Hyperplasia	F	1.5	92.0	x			x
5	Organ Donor	M	1.5	75.0				x
6	Organ Donor	M	2	78.0	x			x
7	Ornithine Transcarbamylase deficiency	M	2	98.2				x
8	Organ donor	F	30	48.0		x	x	
9	Colorectal Cancer	M	31	93.0	x			x
10	Organ donor	F	35	70.0			x	
11	Maple Syrup Urine Disease	M	3	71.0			x	
12	Organ donor	M	45	69.5		x	x	
13	Adenoma	F	50	90.5	x			x
14	Organ donor	M	52	75.0		x	x	
15	Organ donor	F	53	58.0		x	x	
16	Organ donor	F	56	50.1		x	x	
17	Unidentified neoplasia	M	56	77.0			x	
18	Carbamoyl Phosphate Synthetase-1 deficiency	F	0.5	74.5	x			x
19	Colorectal Cancer	F	61	89.8	x			x
20	Unidentified neoplasia	F	66	54.0		x	x	
21	Organ donor	F	68	82.0		x	x	
22	Colorectal cancer	F	68	93.0			x	
23	Colorectal Cancer	F	68	84.3	x			x
24	Hepatocellular Carcinoma	F	72	82.3	x			x
25	Organ donor	F	74	76.0		x	x	
26	Colorectal Cancer	M	78	35.2	x			x
27	Colorectal Cancer	M	79	89.9	x			x
